# Androgen-Induced Immunosuppression

**DOI:** 10.3389/fimmu.2018.00794

**Published:** 2018-04-17

**Authors:** Melanie R. Gubbels Bupp, Trine N. Jorgensen

**Affiliations:** ^1^Biology Department, Randolph Macon College, Ashland, VA, United States; ^2^Department of Inflammation and Immunity, Lerner Research Institute, Cleveland Clinic Foundation, Cleveland, OH, United States

**Keywords:** androgen, testosterone, systemic lupus erythematosus, rheumatoid arthritis, multiple sclerosis, cancer, autoimmunity, sex hormones

## Abstract

In addition to determining biological sex, sex hormones are known to influence health and disease *via* regulation of immune cell activities and modulation of target-organ susceptibility to immune-mediated damage. Systemic autoimmune disorders, such as systemic lupus erythematosus, rheumatoid arthritis, and multiple sclerosis are more prevalent in females, while cancer shows the opposite pattern. Sex hormones have been repeatedly suggested to play a part in these biases. In this review, we will discuss how androgens and the expression of functional androgen receptor affect immune cells and how this may dampen or alter immune response(s) and affect autoimmune disease incidences and progression.

## Introduction

Sex hormones exert their effects on many cellular targets, including cells of the immune system. Indeed, sex hormones directly influence immune cell function and development as well as the susceptibility of cells and tissues to damage from aberrant (autoimmune) processes. In this review, we will discuss how androgens and the androgen receptor (AR) affect immune cells and how this may dampen or alter immune response(s) to affect disease incidence and progression.

## Androgen and ARs

### Androgens

The four androgen hormones, dihydrotestosterone (DHT), testosterone, androstenedione, and dehydroepiandrosterone (DHEA), are all synthesized from cholesterol in the gonads and adrenal glands (1). DHT is more potent than testosterone, while androstenedione and DHEA exhibit only 10 and 5% of the potency of testosterone, respectively (1). However, testosterone is the most concentrated androgen in adult male serum, with DHT present at one-tenth the concentration of testosterone. Testosterone can be converted to androstenedione (and *vice versa*) and both can be aromatized to estrogens by aromatase (2). Aromatase is widely expressed and thus studies in which testosterone and androstenedione have been used for *in vivo* treatment can be difficult to interpret. DHEA binds several steroid hormone receptors, including AR and estrogen receptors α and β, albeit with lower affinity than their cognate ligands (3). Moreover, DHEA can be reversibly modified to form DHEA-S, which can be peripherally metabolized to testosterone (especially in premenopausal women) and estrogens (especially in postmenopausal women) (3), further complicating our understanding of DHEA-mediated effects. Of the four androgens, only DHT cannot be converted to estrogens and thus, studies utilizing DHT are most easily interpreted.

### Androgen Receptors

Beyond its required role in the development and expression of male phenotypes, the AR regulates immune function *via* modulating the transcription of a number of genes by DNA-binding-dependent and -independent mechanisms (4). Encoded on the X chromosome, the AR is a signal transduction protein and transcription factor required for the development and expression of male phenotypes (4). The AR is bound by heat shock proteins and chaperones in the cytoplasm until bound by its ligands (5–10). Signal transduction through the classical AR is a multi-step process dependent upon receptor dimerization, the binding of ligand, interaction with cofactors, and DNA binding. Upon binding ligand, heat-shock proteins and chaperones are exchanged for cofactors, and the receptor:ligand complex translocates into the nucleus to bind specific DNA regulatory sequences [androgen response elements (AREs)] and regulate transcription (4). Due to the differences in binding affinities and dissociation constants, AR:DHT complexes remains bound to AREs longer than AR:testosterone complexes, further adding to the increased potency of DHT (11–13).

In addition to its well-characterized ability to function as a tran-scription factor as outlined above, the AR also signals through DNA-binding-independent mechanisms and can even signal in a ligand-independent fashion (14). Activation of non-classical (NC) AR rapidly affects the regulation of other nuclear receptors, transcription factors, and cytoplasmic signaling events including the release of intracellular calcium and the formation of inositol 1,4,5-triphosphate (15). NC receptors may be located in the plasma membrane, where they are associated with G-protein coupled receptors and subject to internalization, or in the cytoplasm (16, 17) [reviewed in Ref. (18, 19)]. NC ARs include receptors that bind androgen either directly or indirectly *via* the steroid hormone binding globulin (SHBG) (20, 21). Finally, in the context of cancer, AR may be activated by a variety of growth factors independently of androgens (14).

Polymorphisms in the AR gene, *NR3C5*, are known to influence androgen signaling strength. The most widely studied polymorphism affects the number of CAG repeat sequences in exon one of the AR gene. Specifically, AR’s transactivational activity decreases with the presence of longer CAG repeats and *vice versa* (22, 23). Interestingly, women with shorter CAG repeats (i.e., those with more potent AR signaling) exhibit higher androgen levels, while men with shorter CAG repeats experience more dramatic reductions in testosterone as they age (24, 25), suggesting that CAG repeats differentially affect AR signaling in men and women.

The expression of AR in various immune organs and multiple immune cells provides some indication of the level at which androgens influence immunity (Table 1). For example, T cells are sensitive to androgens throughout development and beyond, while B cells are primarily sensitive during development. Indeed, thymocytes and thymic epithelial cells express intracellular AR (26–28) as do peripheral T cells, which also express NC, membrane associated receptors (17, 28). Bone marrow stromal cells and B cell precursors, but not peripheral B cells, express AR (29–31). Gene expression studies show that the AR is expressed by all myeloid progenitor cells as well as some terminally differentiated cells of myeloid lineage, including neutrophils, monocytes, and macrophages (16, 32–36). Thus, there is great potential for androgen modulation of the development and function of both the lymphoid and myeloid branches of the immune system.

**Table 1:** Expression of androgen receptor (AR) in hematopoietic cells.

Cell type	AR expression (method)	Reference
**Stem cells and progenitor cells**		
Hematopoietic stem cell	Yes (RT-PCR, IF)	(33, 34)
Common myeloid progenitor	Yes (RT-PCR)	(33)
Common lymphoid progenitor	Yes (RT-PCR)	(33)
Granulocyte-macrophage progenitor	ND	
Common dendritic cell (DC) progenitor	ND	
Megakaryocyte-erythroid progenitor	ND	
Erythroblast	Yes (binding assay)	(32)
Early T cell progenitor	ND	

**Myeloid-derived cells**		
Megakaryocyte	Yes (IHC, RT-PCR, IF)	(37, 38)
Platelet	Yes (IF)	(38)
Erythroid cell (nucleated and enucleated)	*Not expressed* (IHC)	(37)
Monocyte	Yes (RT-PCR)	(37)
Macrophage	Yes (C+NC) (flow, IF, IHC, RT-PCR)	(16, 36, 37, 39)
Myeloid-derived DC	*Not expressed* (WB)	(40)
Myelocyte	Yes (IHC)	(37)
Metamyelocyte	Yes (IHC)	(37)
Neutrophil (band cell)	Yes (IHC)	(37)
Neutrophil (segmented)	Yes (IHC)	(37)
Mature eosinophil	*Not expressed* (IHC)	(37)
Basophil	ND	
Mature mast cell	Yes (ImmGold)	(35, 41)

**Lymphoid-derived cells**		
DN T cell	Yes (binding assay)	(42)
DP T cell	Yes (binding assay)	(42)
CD4+ T cell	Yes (C+NC) (flow, IF, binding assay)	(17, 28, 42)
CD8+ T cell	Yes (C+NC) (binding assay)	(17, 42)
Plasmacytoid DC	ND	
Pro-B cell	Yes (WB)	(43)
Pre-B cell	Yes (WB)	(43)
Immature B cell	ND	
Mature B cell	*Not expressed* (IHC, WB)	(37, 43)

**Other**		
Thymic epithelial cells	Yes (IF)	(44)
Bone marrow stromal cells	Yes (IHC, WB)	(37, 43)

*ND, not determined; C, classical; NC, non-classical. ImmGold, ImmunoGold staining; RT-PCR, reverse-transcriptase-polymerase chain reaction; WB, western blotting; IF, immunofluorescence; IHC, immunohistochemical staining; flow, flow cytometry*.

## Androgens and Immune Cell Subsets

There is ample evidence that androgens alter immune cell development and immune activation. In the following section, we review the effect of androgens on specific immune cell subsets and the potential effect this may have on immune responses and immune homeostasis in general.

### Myeloid Cells

The innate immune system consists of a number of different cell subsets, predominantly of myeloid origin. Most myeloid cells initiate their track of differentiation from hematopoietic stem cells in the bone marrow only to undergo final differentiation at sites of infection or inflammation. As mentioned above, all myeloid progenitor cells express the classical AR (34, 35) and testosterone has been shown to affect early myelopoiesis (45–47). Myeloid cell-specific effects of androgens are further discussed below.

#### Neutrophils

Several lines of evidence suggest that androgens directly promote the differentiation of neutrophils from myeloid progenitors. For example, both genetically manipulated AR-deficient mice and androgen insensitive mice carrying the naturally occurring testicular feminization mutation (*tfm*) exhibit neutropenia (45, 46). Similarly, androgen-deficient prostate cancer patients and gona-dectomized male mice also display neutropenia, prior to androgen-replacement therapy/DHT treatment (48, 49). Further support for androgen-induced granulopoiesis and neutrophil differentiation comes from studies of stanozolol, a testosterone analog, showing an increased prevalence of myelocytes and metamyelocytes as well as accelerated neutrophil maturation in treated female mice (50). Neutrophilia can also be observed in young women with hyperandrogenism due to polycystic ovarian syndrome (47). Interestingly, treatment with the anti-androgen, flutamide, and metformin (known to reduce circulating levels of androgens) decreases numbers of neutrophils in these patients (47). Together, these studies suggest that androgens drive neutrophil differentiation and/or survival in mice and humans.

Although, less well understood, there is growing evidence that androgens might also affect neutrophil function. For example, testosterone suppresses both the anti-microbial activity and the production of pro-inflammatory cytokines by human neutrophils, while promoting the production of the anti-inflammatory cytokine IL-10 (51, 52). It is interesting that there are no reports of elevated numbers of myeloid cells in athletes taking anabolic androgenic steroids (AAS) as performance enhancing drugs, although increased production of pro-inflammatory cytokines (IL-1β and TNFα) and greater oxidative stress responses in PBMCs from AAS users [reviewed in Ref. (53, 54)] do suggest an effect on myeloid cell activity. In summary, androgens appear to promote neutrophil differentiation in mice and humans and may also dampen the inflammatory potential of mature neutrophils.

#### Monocytes/Macrophages

Both monocytes and macrophages have been found to express classical as well as NC AR (16, 37, 39, 41). Testosterone treatment was shown to elevate levels of circulating monocytes in a population of type II diabetic men with partial androgen deficiency (55), however, whether this effect was due to augmented differentiation of monocytes in the BM or altered trafficking patterns remains unknown. Studies evaluating the importance of androgens and/or AR in macrophages during wound healing have shown that AR deficiency accelerates wound healing, while DHT treatment improves the quality of the wound by increasing collagen fibers (56, 57). It remains to be determined at which stages of wound healing testosterone/DHT binding to the AR is required, which may explain these seemingly contradictory results. Finally, gonadectomy has been found to drive increased TLR4 expression by male murine macrophages leading to elevated pro-inflammatory responses during infection (58), suggesting that one mechanism by which androgens are immunosuppressive is by limiting myeloid cell responsiveness to pathogens. This observation is further supported by data showing higher TLR4 expression, increased phagocytosis, and enhanced oxidative burst in female macrophages (59) and a specific downregulation of TLR4 expression by testosterone *in vitro* (58). Interestingly, male mice subjected to sepsis fare worse than females (60), although whether the outcome is dependent on testosterone-mediated suppression of myeloid cell activity remains unknown.

At the molecular level, studies have identified the presence of plasma membrane-located G-protein receptor coupled NC ARs on macrophages. These receptors are capable of binding testosterone either directly or bound to SHBG and elicit non-transcriptional stimulatory effects, such as rapid intracellular calcium mobilization and ERK phosphorylation (16, 20, 21). More research is needed to fully understand the impact of NC AR activation on macrophage function in males and females.

#### Other Myeloid-Derived Cell Subsets

##### Mast Cells

Skin residing mast cells have been found to express the AR, however, neither numbers nor distribution of these cells are affected by altering levels of androgens (35, 41). Instead, mast cell function, as determined by histamine release, may be regulated by androgens, as histamine levels at some peripheral sites are reduced after castration (61). More recently, it was shown that at least under some circumstances testosterone directly induces *Il33* expression by mast cells (62). Interestingly, IL-33 drives the generation of both innate lymphoid cells and basophils, known to produce Th2-associated cytokines and promote antibody class switching to IgE; an immunoglobulin found to be increased in young males over females in individuals suffering from allergic rhinitis (63, 64). Future studies will determine if androgens drive activation of other mast cell-specific proteins and processes.

##### Eosinophils

In contrast to neutrophils, numbers of eosinophils increased in the periphery and in the bone marrow of gonadectomized male mice (65). Castration did not, however, affect eosinophil numbers within sites of exposure, as eosinophil counts in nasal mucosa of unmanipulated and castrated male mice challenged with phospholipase A2 and *Schistosoma mansoni* egg antigen were comparable (66). Interestingly, testosterone directly reduced human eosinophil viability and adhesion properties *in vitro* (67), and DHEA suppressed eosinophil trafficking to the lung during infection (68), suggesting that androgens affect eosinophil numbers *via* control of tissue infiltration rather than *de novo* differentiation in the bone marrow. Additional studies evaluating the effect of the non-aromatizable DHT are needed to firmly address the role of AR and androgens on eosinophil maturation and function.

##### Basophils

Basophils are largely unaffected by testosterone treatment (69) and expression of AR by these cells has not been determined.

### Dendritic Cells (DCs)

At the border of the innate and the acquired immune systems, reside DCs. These can originate from either myeloid or lymphoid progenitors. Only few studies have investigated the effect of androgens on DC differentiation and function but overall, testosterone has been assigned an immunoinhibitory function. It remains controversial, however, whether this effect is direct or indirect, as at least one study demonstrated a lack of AR expression in myeloid-derived DCs (40). Nevertheless, exogenous DHT treatment has been found to either downregulate surface levels of MHC/HLA and costimulatory molecules or inhibit cytokine production in animal models resulting in reduced T cell activation, proliferation, and differentiation. For example, after LCMV infection, infiltrating DCs isolated from the brains of male mice were less activated (reduced MHC-II and CD86 expression) than cells isolated from females and gonadectomized male mice (70). This observation was due to testosterone, as DHT treatment of gonadectomized male mice reversed the DC phenotype back to that of intact males (70). Similarly, gonadectomy studies have shown that removal of testicular testosterone production in male mice results in increased expression of MHC and costimulatory molecules on DC (71). A similar pattern is found in hypogonadal men, in whom DC activation markers are significantly elevated, but reversed in response to exogenous testosterone treatment (72). The *in vivo* nature of these experiments and observations, however, do not necessarily support a direct effect of androgens on DCs, as both MHC and costimulatory molecules are also regulated by cytokines secreted by other cells subject to androgenic regulation. Specifically addressing this concern, bone marrow-derived DCs, exposed briefly to DHT during antigen uptake, have been found to be less efficient T cell activators *in vitro* than BMDCs not exposed to DHT (73).

### Lymphoid Cells

#### B Cells

It has been known for decades that the average female produces higher levels of antibodies in response to infections and vaccinations [reviewed in Ref. (74)]. A number of studies have found strong correlations between low testosterone and elevated numbers of B cells (75–79), and high testosterone levels in men correlates with poorer antibody responses to vaccination (80), suggesting that androgens inhibit B lymphopoiesis. Recent studies of B cell subsets in 3- to 8-year-old children identified different distributions in males and females (81, 82). Specifically, boys demonstrate elevated levels of immature CD5+ B cells, while girls exhibit more memory-type B cells. Lundell et al. further evaluated levels of DHT in these children and found a positive correlation between DHT levels at birth and the frequency of immature B cells. Given the minimal exposure to exogenous agents, these data suggest that males and females are subject to differential genetic- and/or hormonal-driven gestational regulation of B cell lymphopoiesis.

In mouse studies, gonadectomy of male mice has been found repeatedly to drive B cell lymphopoiesis in the bone marrow, with both testosterone and DHT treatment capable of reversing this effect (43, 83, 84). Similarly, global AR-deficient mice present with elevated B cell precursors in the bone marrow (pro-B stage and beyond) and studies of the B cell repertoire in B cell-specific AR knockout animals suggest that the lymphopoietic effect of testosterone is AR-dependent and intrinsic to the B cell—at least at the later stages of B cell development (pre-B cells and beyond) (85). However, other studies have suggested that the inhibitory effect of testosterone on B lymphopoiesis is dependent on bone marrow stromal cells (29, 30). Recently, AR expression by bone marrow osteoblasts was found to specifically inhibit early B lymphopoiesis resulting in an accumulation of pro-B cells (86). Thus, it is likely that the differentiation of pro-B cells from common lymphoid progenitors is inhibited by AR expression by osteoblasts, while further differentiation along the B cell lineage is negatively affected by AR expression by the B cell progenitors themselves. A possible mechanism of action has been suggested based on studies showing that testosterone upregulates TGFβ production by bone marrow stromal cells leading to inhibition of IL-7 production and suppression of B lymphopoiesis (29, 87). In summary, B lymphopoiesis is inhibited by androgens both directly and indirectly *via* effects on bone marrow stromal cells.

#### T Cells

Thymic size and the selection of developing thymocytes is signi-ficantly affected by androgens. Testosterone deficient or insensitive males, due to gonadectomy or AR deficiency, experience thymic enlargement (27, 88–93). Thymic size returns to normal when gonadectomized males are treated with DHT (91). Studies involving AR-deficient bone marrow chimeric mice demonstrated that androgen signaling through AR in thymic epithelial cells mediates androgen’s effects in the thymus (27). A similar observation was made in thymic epithelial cell-specific AR−/− mice (94). In addition, androgens limit the numbers of CD4+ CD8+ and CD4+ CD8− in favor of CD4− CD8+ thymocytes, perhaps by suppressing proliferation and accelerating the apoptosis of immature thymocytes (88, 90, 95, 96). Finally, androgens enhance the negative selection of self-reactive T cells by upregulating the expression of *autoimmune regulator* (*Aire*) in medullary thymic epithelial cells (97). Androgens may also influence T cell development in tolerance-promoting ways by enhancing TGF-β production in the thymus (98).

Similar to its effect on thymic size, androgens also limit the total number of T cells residing in the periphery. Postpubescent gonadectomized male mice exhibit larger peripheral lymphoid organs housing a greater number of lymphocytes, including both CD4+ and CD8+ T cells (92, 99). The expansion of peripheral lymphoid organs after the removal of androgens may be related to increases in thymic output and/or lessened peripheral T cell death, as an *in vitro* study recently demonstrated that DHT can non-selectively induce cell death in peripheral T cells (100).

Androgens are likely responsible for some portion of the effect of sex on peripheral T cell responses. Thymocytes and lymphocytes isolated from non-autoimmune female mice respond more vigorously to exogenous and allogeneic antigens *in vitro* than cells isolated from male mice (101, 102). Treating female mice with testosterone reduces the proliferative T cell response to OVA and KLH (101). Similarly, gonadectomized male mice, compared to intact male mice, proliferate more vigorously in response to TCR stimulation and OVA *in vitro* and *in vivo* (92, 99).

Cytokine production evoked by specific antigens are also often affected by sex, with male cells favoring Th2 type responses and female cells favoring Th1 type responses (92). For example, anti-CD3 stimulation provokes the secretion of Th2 cytokines, IL-10 and IL-4, from CD4+ T cells isolated from male experimental autoimmune encephalomyelitis (EAE)-prone SJL mice, but IL-12 from cells isolated from females (26). Interestingly, the addition of DHT to T cell cultures is sufficient to upregulate IL-10 expression (26). Beyond its ability to enhance the production of Th2 cytokines, androgens actively inhibit Th1 differentiation (103, 104), by inhibiting IL-12 and IFN-γ production downstream of antigen stimulation (105–109).

Androgens may influence the differentiation and function of regulatory T cells differently in males versus females. *In vivo* androgen supplementation of women with adrenal insufficiency and female rats with experimental autoimmune orchitis expands the number of regulatory T cells (104, 110). More specifically, when bound to ligand, the AR directly enhances the expression of *Foxp3* in T cells (regulatory or otherwise) isolated from rats or women during the ovulatory phase, but not men (111). Thus, androgens are capable of directly converting peripheral T cells into regulatory T cells in women. By contrast, androgens may interfere with regulatory T cell function in men, as occurs in a mouse model of Sjögren’s syndrome that predominantly affects male mice (112).

Overall, androgens directly influence the development of lymphoid cells; and at least in mice, lymphoid cells that develop in the presence of androgens may retain differential characteristics even when later placed in an androgen-deficient environment. Moreover, androgens appear to suppress the inflammatory potential of peripheral lymphoid cells. In some cases, such effects may be direct, but the absence of AR in peripheral B cells, for example, suggests that differences are more likely due to prior exposure to androgens during development, or regulation by other androgen-sensitive peripheral cells.

## Androgens in Autoimmunity

Many autoimmune disorders are more prevalent in females, including autoimmune thyroiditis, systemic lupus erythematosus (SLE), Sjögren’s syndrome, multiple sclerosis (MS), and rheumatoid arthritis (RA) (113). Both sex hormones and genes expressed on the X or Y chromosomes have been proposed to drive this bias, as exemplified by the fact that Klinefelter’s patients (XXY karyotype) express not only two X chromosomes but also reduced levels of androgens, and present with an increased risk for most of these disorders (114). Many patients of either sex with autoimmune disorders that predominantly affect women also demonstrated lower serum concentrations of androgens (76, 115, 116). Here, we will discuss the influence of androgens on the development and severity of RA, MS, and SLE.

### Rheumatoid Arthritis

Rheumatoid arthritis is characterized by synovial inflammation and swelling, as well as cartilage and bone destruction. Some patients may develop one or more additional systemic sequelae, including cardiovascular disease, pulmonary disorders, lymphoma, lung cancer, psychological disorders, and osteoporosis (117). Like many other autoimmune diseases, RA is 2–4 times more frequent in women than in men (113). Most studies have concluded that in addition to increased susceptibility, female RA patients suffer from a more severe version of the disease, with higher disease activity scores, faster progression, more pain, and lower remission rates (118–122).

While estrogens likely contribute to the increased female risk of RA (123), it has been hypothesized that androgens may also offer some protection from the disease. Indeed, serum androgen levels tend to be lower in men with RA as compared to healthy controls. For example, the incidence of RA increases as androgen production declines in aging men and several groups have reported lower serum testosterone concentrations in male RA patients as compared to controls (76, 124–130). Furthermore, men who experience a dramatic loss of serum androgens with age may develop a more aggressive form of RA with earlier onset (131–133). In some cases, low serum androgens also correlate with increased risk of developing RA. Men with genetic hypogonadism (Klinefelter’s syndrome) and prostate cancer patients treated with androgen-ablating therapy are at increased risk of developing RA (114, 134).

Interestingly, serum androgen levels are not lower prior to the onset of RA in all patients, suggesting that low androgens are not universally predisposing to the development of RA (127, 133). Instead, as a recent large study reported, men with lower serum testosterone levels prior to the onset of RA may be more likely to develop a specific subset of RA, characterized as rheumatoid factor negative (135). It is possible that the correlation of low serum androgen levels and RA in men can be explained by inflammatory cytokines, such as IL-6, which become elevated during the disease process and are known to suppress the secretion of adrenal androgens (136). The notion that low serum androgens are a consequence of RA, as opposed to a cause in some cases, is supported by an inverse correlation between low free testosterone and inflammatory markers and disease activity (129, 137), as well as the finding that successful treatment of RA correlates with the restoration of normal levels of free testosterone (130). To summarize, androgens may protect against the development of RA in men in some circumstances; and in others, the inhibition of androgen secretion by the RA-inflammatory response is secondary to RA, but may still influence the severity of disease in men.

The effect of androgens on RA susceptibility and severity in women is less well understood. Androgens may protect against RA in some women, but other studies suggest that androgens may actually worsen disease severity. As in men, low serum concentrations of androgens, particularly DHEA and/or DHEA-S, are linked with RA in women (136, 138–140). However, as was found in men, serum androgen levels are within the normal range 10 years prior to the onset of RA and levels of DHEA-S inversely correlate with disease duration and severity in women (127, 140). Thus, for most female patients, the inhibitory effect of inflammation on the secretion of androgens may explain this correlation. Two notable exceptions to this include women who inherited particular polymorphisms resulting in higher or lower androgen levels correlating with protection from disease or exacerbation, respectively. First, women predisposed to produce androgens in greater concentrations due to inheritance of a polymorphism in the CYB5A gene are protected from developing RA characterized by RF and antibodies to citrullinated proteins (141); and, second, women with lower serum androgens are more likely to develop a type of RA that is not responsive to combination therapy consisting of nonsteroidal anti-inflammatory drugs, low-dose prednisolone, methotrexate, and more than one of several other disease modifying anti-rheumatic drugs (142).

In contrast to the above findings, high androgen concentrations or more potent AR signaling have been reported in some women with more severe RA. For example, one small study reported normal androgen concentrations in premenopausal RA patients, and higher testosterone and DHEA-S in postmenopausal women with RA (143). More strikingly, women with higher concentrations of serum androgens due to a low number of CAG repeats in the AR developed a more aggressive RA with earlier onset, though overall susceptibility to RA was not affected (131–133, 144).

Because of the correlation between low serum androgens and RA and the known immunosuppressive properties of androgens, androgens have been utilized to some extent as a treatment for RA. Overall, the administration of androgens to male and female patients had a positive effect for both sexes (145–148). However, it should be noted that such studies are few in number, with small patient populations, short-term treatments, modest improvements, and in some cases, no effect at all.

#### Animal Models of RA

Several animal models of RA also show increased susceptibility or more severe disease in females as compared to males (149–152). For example, the incidence of RA is greater or more severe for females in collagen-induced arthritis (CIA) in rats, SKG mice injected with zymosan, LEW/N rats injected with polysaccharide fragments from group A streptococcal bacteria, and BALB/c mice with cotton-pellet induced inflammation (149–152). Moreover, androgens have been shown to exert protective effects in RA, even in animal models with equal or more severe disease in males (150, 153). Gonadectomy of male animals worsens RA in CIA-rats and SKG mice; and, the addition of DHT to gonadectomized CIA-rats inhibits disease (149, 152). Male and female rats injected with complete Freund’s adjuvant (adjuvant arthritis) do not demonstrate a sex bias; however, similar to human studies, arthritic males demonstrate lower testosterone levels after disease induction (153). Finally, the expression of the AR on B cell progenitors has been shown to have protective effects in male mice with CIA (150).

#### Cellular and Molecular Targets of Androgens in RA

Regardless of whether serum androgen levels or receptor activity is involved in systemic RA etiology, a separate case has been made for its involvement in disease pathogenesis within affected joints. The synovial fluid of RA patients exhibits elevated levels of free estrogens and reduced concentrations of free androgens, possibly due to increased local aromatization of androgens to estrogens (154). The conversion of androgens to estrogens heightens the local inflammatory response, since androgens have been shown to inhibit the synthesis and secretion of IL-1 and IL-6, two important inflammatory cytokines in RA (155–158). The relationship between androgens, inflammatory cytokines, and aromatase activity is reciprocal; IL1 and IL6 stimulate aromatase activity, while androgens inhibit it (157).

In summary, with some notable exceptions, the correlation bet-ween low androgen and RA likely exists because RA-associated inflammation dampens serum androgen levels. Improvements in our ability to group RA into less heterogenous disease subgroups may reveal particular subgroups that are more affected by androgen levels than others.

### MS and Androgens

Multiple sclerosis is an autoimmune disorder in which neuronal axons are actively demyelinated leading to neuronal damage and eventual paralysis. The disease precipitates in patterns of relapsing-remitting or progressive-onset. Only the former of these shows a sex-bias; relapsing-remitting MS (RRMS) develops 3–4 times more frequently in females than in males and predominantly in individuals of childbearing age, suggesting a role for sex hormones [reviewed in Ref. (159)]. In addition to higher incidence rates among female RRMS patients, many studies have also shown that women exhibit higher relapse rates than men (160–165), further supporting a gender-bias in this disease. Several studies have evaluated levels of sex hormones in MS patients, and testosterone, DHEA, or DHEA-S levels have been found to be lower in both men and women with MS as compared to healthy age-matched controls (115, 165–168). Although it is generally thought that testosterone’s protective effect is mediated *via* immune modulation, treatment with testosterone improved cognitive performance and slowed brain atrophy (169) and has been suggested to increase gray matter in a small cohort of men with RRMS, suggesting a direct neuroprotective function of testosterone (170).

Multiple sclerosis patients display a chronic inflammatory profile characterized by T cell-derived cytokines (IFNγ and IL-17) and circulating antibodies reactive to brain autoantigens (171–176). Both T cell and B cell differentiation and effector functions have been shown to be affected by androgens (as mentioned above), however, clinical trials with drugs specifically targeting IL-17A (secukinumab) or B cells (rituximab) were only somewhat effective (177, 178), and targeting IFNγ *via* blockade of IL-12 (ustekinumab) was not effective (179).

#### Animal Models of MS

Studies of EAE (animal model of MS) have largely confirmed a protective role for androgens (180–183). For example, gonadectomy of male SJL mice resulted in increased disease susceptibility, while treatment with exogenous testosterone or DHT reduced disease incidence in both females and males (180, 181). Although C57Bl/6 male and female mice develop EAE at a similar rate, C57Bl/6 male mice were also protected by treatment with DHT (181), and similar results have been obtained in EAE-susceptible Dark Agouti rats (183). By contrast, Ziehn et al. showed that only testosterone, not DHT, exerted a direct neuoprotective effect (182), suggesting that testosterone and DHT may have independent effects on cells of the hippocampus and infiltrating immune cells. Further studies are needed to thoroughly investigate if AR binding is required for the protective effect of androgenic treatment.

#### Cellular and Molecular Targets of Androgens in MS/EAE

While most studies support an overall protective effect of testosterone in MS and EAE, specific immunological targets are less well explored. Androgens may affect T cells in at least two specific ways. As mentioned above, androgens stimulate the *Aire* promoter, driving increased Aire expression by medullary thymic epithelial cells and increased negative selection (97). As a result hereof, male mice or mice treated with DHT are relatively protected from EAE (97). Second, EAE is typically driven by T helper cells (Th1 and Th17) and is characterized by the presence of key signature cytokines such as IFNγ and IL-17 within the brain, secondary lymphoid organs, and circulation. A general Th1 propensity has been observed in female patients with MS and EAE animal models (184–187) and it has been suggested that low levels of testosterone drive this phenotype. In support hereof, *ex vivo* exposure of encephalomyelitic T cells to testosterone has been shown to significantly change the secreted cytokine profile (from IFNγ to IL-10) and the pathogenic potential of these T cells (180, 188). Furthermore, myelin-basic protein-primed female T cells and T cells from gonadectimized males express significantly higher levels of the VLA-4 integrin β1 subunit and secrete higher levels of pro-inflammatory cytokines, such as IL-1β, than male-derived cells (189), thereby promoting T cell infiltration into the brain and brain pathogenesis. Although the mechanism driving differential T cell activation in males and females is largely unknown, Dunn et al. recently described that PPARα was highly expressed in male T cells in a testosterone-dependent manner and that deficiency of PPARα specifically worsened EAE in male mice (109). Further studies are needed to establish the interrelationship between PPARα, Aire, and other DHT-dependent immune regulators.

In conclusion, low levels of androgens are observed in patients with MS and gonadectomy of male mice increases their susceptibility to induced EAE. T cells have been found to respond to androgens throughout development and recent studies have started to unravel molecular mechanisms associated with androgen-induced T cell suppression.

### Androgens in SLE

Systemic lupus erythematosus, a chronic and potentially fatal disease with the potential to cause damage in multiple organ systems, is nine times more prevalent in women than men (190). Physicians commonly see patients with a wide range of clinical manifestations, which may spontaneously flare and remit. For example, patients with mild lupus may present with intermittent skin rash and joint pain and require little medication, while patients with severe glomerulonephritis may show progressive renal deterioration despite treatment with high doses of corticosteroids and cytotoxic drugs. Other significant health consequences can include central nervous system involvement, vasculitis, thrombosis, thrombocytopenia, anemia, fevers, fatigue, and heart and lung involvement (190, 191).

Antinuclear autoantibodies (ANAs) are generally considered to be a hallmark of lupus (190, 191). A portion of the tissue damage in SLE is related to autoantibodies that target cell surface antigens. In other cases, such as in the kidney, the deposition, or *in situ* formation of ANA immune complexes with subsequent complement activation and inflammatory cell recruitment are responsible for the damage. The damage generated by immune complexes is not trivial; SLE is a leading cause of kidney disease, stroke, and premature cardiovascular disease in young women (192, 193).

A number of reports have suggested that androgens are protective in SLE. Although many male lupus patients have normal levels of androgens (194), men with hypogonadism are at increased risk of developing SLE (114, 116, 195) and testosterone supplementation of male lupus patients with genetic hypogonadism (Klinefelter’s syndrome) has, in two cases, been beneficial in treating lupus (196, 197). However, no large-scale studies involving testosterone supplementation in male lupus patients have been reported (198). Women with lupus generally have lower androgen levels, including testosterone, DHT, DHEA, and DHEA-S (199–201), and demonstrate an accelerated inactivation of testosterone *via* oxidation than healthy age-matched controls (202). In addition, it has been suggested that inflammatory cytokines in affected tissues modulate aromatase activity to locally dampen the effects of androgens in favor of estrogens in lupus patients (203). Finally, male lupus patients with reduced AR signaling produce higher quantities of IgG autoantibodies (204), while female lupus patients with the same polymorphism exhibited reduced disease (78). It is not clear why reduced androgen signaling is associated with opposite effects on lupus in male and female patients. However, recent studies in the BWF1 animal model (discussed below) have also identified instances whereby androgen-mediated mechanisms of disease suppression are not effective in females (48). Together these observations suggest that androgens affect lupus pathogenesis differently in males and females and that the mechanisms by which androgens limit disease may not be immediately applicable as therapies for female patients.

Although survival rates of male lupus patients are comparable to survival rates in female lupus patients (205–210), it has been reported that the severity of SLE is worse in males than in females, suggesting that genetic susceptibility must be more potent in men to overcome the protection afforded by androgens (211–215). This is supported by reports that male lupus patients have an increased frequency of renal involvement (208, 210, 216–218) and that women with an affected male relative are 3.5 times more likely to develop renal disease than women without an affected male relative (219).

Some effort has been made to treat lupus with androgenic compounds. The small numbers of patients included in many of the following clinical trials make definitive conclusions difficult. However, in general, these studies support an ameliorating role for androgens. Danazol, a weakly androgenic synthetic compound, reduced total serum IgG as well as anti-dsDNA autoantibody levels in women (220, 221). However, all patients did not benefit from therapy and some experienced disease flares (220, 221). Like-wise, treatment with a testosterone-like anabolic steroid, nandrolone, afforded some patients clinical improvement, but other patients saw no improvement and masculinizing side effects made this drug a somewhat untenable treatment option for most women (202, 222, 223).

Larger clinical trials have been conducted with DHEA, an adrenal steroid with mild androgenic activity, in the hopes of separating the disease-ameliorating properties of androgens from its masculinizing ones. However, as for other androgenic drugs, some patients treated with DHEA (also known as prasterone) experienced reduced disease activity, improved cognitive function, enhanced mental well-being, and a reduced need for corticosteroid treatment (224–228), while others experienced no improvement (229, 230). Overall, treatment with this agent did not meet primary objectives of these studies and it has not been approved by the Federal Drug Agency for treatment of lupus patients. It should be mentioned that in a recent study, female lupus patients with a particular polymorphism in the extra-pituitary prolactin gene associated with low serum DHEA levels experienced the most dramatic improvements after DHEA treatment (201). Thus, genetic differences of particular study populations may explain the varied results from different clinical trials with DHEA in lupus patients.

#### Androgens in Mouse Models of Lupus

Inbred mice that spontaneously develop a lupus-like disease have been extremely helpful toward elucidating the etiology and pathogenesis of lupus. Several spontaneous models of lupus exist [reviewed in Ref. (231)]. Here, we focus exclusively on studies conducted with the F1 offspring of New Zealand black (NZB) and New Zealand white (NZW) mice. The female F1 offspring of NZB and NZW mice (BWF1) develop a lupus-like disease characterized by high levels of IgG ANAs accompanied by a severe and progressive glomerulonephritis in the first year of life (232, 233). By contrast, less than half of BWF1 male mice develop severe proteinuria within the same time period (232, 233). As in humans, several studies have determined that androgens suppress lupus pathogenicity BWF1 mice. For example, prepubescent gonadectomy of BWF1 male mice increases the incidence of proteinuria and mortality and accelerates the appearance of ANAs as compared to intact males (233–235). In addition, administration of DHT to gonadectomized male mice is sufficient to reduce disease development comparable to that observed in intact male mice (233–235).

Chemical manipulation of androgens in lupus-prone BWF1 mice also generally supports the hypothesis that androgens are protective in lupus. For example, treatment of BWF1 females with nandrolone decanoate (236–238) and ethylestrenol (239), which are both testosterone-like anabolic steroids, ameliorated disease. Nandrolone decanoate also reduced IgG anti-dsDNA antibody, reduced the incidence of proteinuria, and improved survival (236, 238). Similarly, DHEA treatment significantly delayed disease onset, reduced IgG anti-dsDNA autoantibodies, and reduced mortality (240). By contrast, danazol did not protect BWF1 females from accelerated development of disease (241), and treatment of BWF1 females with the anti-androgenic drug, flutamide, resulted only in a slight decrease in survival, with no noticeable effect on autoantibody levels (242). Overall, BWF1 male and female mice recapitulate much of the sex bias observed in lupus patients and are a useful model for advancing our understanding of the role of androgens in lupus-like disease.

#### Cellular and Molecular Targets of Androgens in Lupus

Many autoimmune diseases that are more prevalent in females, including SLE, are characterized by increased numbers of B cells and circulating autoantibody levels [reviewed in Ref. (243)]. Some evidence suggests that androgens may indirectly regulate isotype switching from IgM to more pathogenic IgG autoantibodies in BWF1 mice. Serum testosterone levels dramatically drop in intact BWF1 males at 9 months of age (234), paralleling the time at which autoantibodies in intact males class switch to IgG (233, 244). Furthermore, treating 9-month-old BWF1 males with physiological levels of DHT greatly decreases mortality, prevents IgM anti-polyA antibodies from class switching to IgG, and reduces the levels of IgG anti-dsDNA antibodies (234). By contrast, DHT treatment in intact BWF1 female mice after autoantibody production (6 months) does not affect levels of IgG anti-dsDNA autoantibodies, although mortality is reduced (245). Though the mechanism by which androgens suppress disease in older mice remains unclear, some studies suggest that androgens enhance (and estrogens delay) immune complex clearance (246); a process frequently associated with the development of SLE and lupus in mouse models. For example, androgens have been shown to enhance serum levels of complement components C4, Slp, C5, C6, and Ss binding protein, which could underlie more efficient IC clearance (247–249). More current studies evaluating a relationship between androgens and complement has to our knowledge not been performed.

As indicated above, androgens have been shown to have discreet effects on B cell function and downstream kidney damage in males and females. Androgens also appear to modulate the development and function of neutrophils differently in lupus-prone males and female. We have previously shown that Gr1+ CD11b+ cells accumulate in male mice, inhibiting the function of T follicular helper cells, germinal center formation, and plasma cell differentiation (48, 49, 250). Interestingly, these myeloid cells suppress disease in male, but not female, mice. It is intriguing to speculate that perhaps the increased frequency of suppressive Gr1+ CD11b+ cells in males is a related to CD11b+ cell overexpression of the DHT-regulated gene, *colony-stimulating factor 3-receptor* (*Csf3-r*) (233). Together with its ligand, G-CSF, CSF3-R is involved in maintaining neutrophil homeostasis and also regulates several aspects of neutrophil function (251). Interestingly, high doses of G-CSF suppress lupus-like disease in at least one animal model (252) and polymorphisms, the *Csf3-r* gene influence the development of lupus and RA (253, 254). Thus, it is not unreasonable to hypothesize that sex-specific alterations in expression of CSF3-R could influence neutrophil phenotype and function and thereby differentially influence lupus pathogenesis in males and females. Overall, at least in mice, it seems as though lupus pathogenesis may proceed in fundamentally different ways in male and female mice.

## Cancer and Androgens

Immune cells must be capable of distinguishing modified-self from self, and yet, must not become unduly activated against unmodified self-antigens. Immune activity outside of these boundaries likely risks the development of cancer or autoimmunity and it follows that immune systems especially good at protecting against cancer may run the risk of triggering autoimmunity (and *vice versa*). Given that women are more susceptible to many autoimmune diseases, one might expect men to be more susceptible to cancer. Indeed, a number of epidemiologic studies and meta-analyses have recently confirmed that cancer develops more frequently in males than in females (255–257). In a meta-study examining mortality rates from 1977 to 2006, the male-to-female mortality-rate ratios were also found to be increased all malignant cancers, although predominantly for cancers affecting the upper gastro-intestinal tract and respiratory systems (lip, tongue, hypopharynx, esophagus, larynx, lung, etc.) as well as liver and bladder (255). Many factors have been proposed to explain this discrepancy including environmental exposures (smoking, obesity, infections) and sex hormone levels and signaling (255, 258).

As mentioned above, chronic inflammation can lead to suppression of testosterone. Cancer is known to induce a stage of chronic inflammation, and thus in order to study a potential causal relationship between testosterone and cancer development, retrospective cohort analyses investigating levels of testosterone *prior to* the diagnosis of cancer are necessary. Not many studies of that kind have been done. In one study, testosterone levels were found to positively correlate with the risk for a subset of epithelial ovarian cancers (259). Similarly, higher levels of testosterone correlated with an increased risk of breast cancer in women (260). It will be interesting to see if this pattern holds for other types of cancer.

### Animal Models of Cancer

More direct evidence supporting a tumorigenic role for androgen in cancer development comes from animal studies, where susceptibility to cancer in response to chemicals and genetic manipulation often depend on sex hormone levels (261). For example, gonadectomy of male rats reduced both chemical-induced pancreatic tumor burden (262) and renal cell carcinoma (263), while re-administration of testosterone at least partly reversed this effect. Furthermore, injection of prostate cancer cells into unmanipulated and gonadectomized nude male mice showed reduced tumor growth in gonadectomized mice and increased tumor growth upon subsequent testosterone treatment (94). It should be noted that whether these effects of testosterone were due to inhibition of immune activation or *via* direct effects on tumor cells remains to be tested. In a separate study of induced thyroid cancer in male mice, gonadectomy led to an upregulation of tumor-suppressor genes (264). With tumor-suppressor proteins present, CCL5 chemokine expression by tumor cells increased driving infiltration by inflammatory macrophages and CD8+ cytotoxic T cells and subsequently reduced tumor growth. Thus, testosterone may be predictive of increased cancer risk, warranting further research into the immunosuppressive and potential cancer-promoting effects of testosterone during early establishment of cancer.

## Concluding Remarks

To conclude, sex-specific biases in autoimmunity and cancer incidence are associated with many differences in immune cell development and function. A significant portion of these differences are the result of exposure to androgens. Androgen-mediated suppression of immune reactivity and inflammation increases the threshold for autoimmunity to develop, but likely lowers the threshold for cancer. Studies further uncovering immune-specific effects of androgens are needed and may lead to the identifi-cation of pathways that could be targeted therapeutically to inhibit the incidence and progression of autoimmunity and cancer.

## Author Contributions

MGB and TNJ contributed equally to this project.

## Conflict of Interest Statement

The authors declare that the research was conducted in the absence of any commercial or financial relationships that could be construed as a potential conflict of interest.
